# Pepsinogen and Serum IgG Detection Is a Valuable Diagnostic Method for *Helicobacter pylori* Infection in a Low-Prevalence Country: A Report from Sri Lanka

**DOI:** 10.3390/diagnostics11081364

**Published:** 2021-07-29

**Authors:** Dalla Doohan, Kartika Afrida Fauzia, Jeewantha Rathnayake, Meegahalande Durage Lamawansa, Langgeng Agung Waskito, Vo Phuoc Tuan, Azzaya Dashdorj, Evariste Tshibangu Kabamba, Bui Hoang Phuc, Shamshul Ansari, Junko Akada, Takashi Matsumoto, Tomohisa Uchida, Takeshi Matsuhisa, Yoshio Yamaoka

**Affiliations:** 1 Department of Environmental and Preventive Medicine, Faculty of Medicine, Oita University, Yufu 879-5593, Japan; doctordoohan@gmail.com (D.D.); kartikafauzia@gmail.com (K.A.F.); langgengaw@gmail.com (L.A.W.); vophuoctuandr@gmail.com (V.P.T.); azzaya2000@gmail.com (A.D.); evaristetshibangu@gmail.com (E.T.K.); buihoangphuc412@gmail.com (B.H.P.); shamshulansari483@yahoo.com (S.A.); akadajk@oita-u.ac.jp (J.A.); tmatsumoto9@oita-u.ac.jp (T.M.); 2 Institute of Tropical Disease, Universitas Airlangga, Surabaya 60115, Indonesia; 3 Department of Public Health and Preventive Medicine, Faculty of Medicine, Universitas Airlangga, Surabaya 60115, Indonesia; 4 Department of Surgery, Teaching Hospital Peradeniya, University of Peradeniya, Kandy 20404, Sri Lanka; jeewanrath@gmail.com (J.R.); mdyasas@yahoo.co.uk (M.D.L.); 5 Department of Endoscopy, Cho Ray Hospital, Ho Chi Minh 749000, Vietnam; 6 Research Center for Infectious Sciences, Department of Parasitology, Graduate School of Medicine, Osaka City University, Osaka 545-8585, Japan; 7 Department of Microbiology, Teaching Hospital, Chitwan Medical College, Bharatpur 44200, Nepal; 8 Department of Molecular Pathology, Faculty of Medicine, Oita University, Yufu 879-5593, Japan; tomohisa@oita-u.ac.jp; 9 Department of Gastroenterology, Tama Nagayama University Hospital, Nippon Medical School, Tokyo 206-8512, Japan; matuhisa@m8.dion.ne.jp; 10 Department of Medicine, Gastroenterology and Hepatology Section, Baylor College of Medicine, Houston, TX 77030, USA; 11 Global Oita Medical Advanced Research Center for Health (GO-MARCH), Faculty of Medicine, Oita University, Yufu 879-5593, Japan; 12 Division of Gastroentero-Hepatology, Department of Internal Medicine, Faculty of Medicine, Dr. Soetomo Teaching Hospital, Universitas Airlangga, Surabaya 60115, Indonesia

**Keywords:** Sri Lanka, *Helicobacter pylori*, prevalence, infectious disease, anti-Hp IgG, gastroduodenal diseases, pepsinogen

## Abstract

The use of serum anti-*Helicobacter pylori* IgG and pepsinogen (PG) detection as a diagnostic method was evaluated in Sri Lanka. Gastric biopsies were performed (353 patients), and the prevalence of *H. pylori* infection was 1.7% (culture) and 2.0% (histology). IgG serology testing showed an area under the curve (AUC) of 0.922 (cut-off, 2.95 U/mL; specificity, 91.56%; sensitivity, 88.89%). Histological evaluation showed mild atrophy (34.3%), moderate atrophy (1.7%), metaplasia (1.7%), chronic gastritis (6.2%), and normal tissue (56%). The PGI/PGII ratio was significantly higher in *H. pylori-*negative patients (*p* < 0.01). PGII and PGI/PGII levels were lower in patients with metaplasia than in those with normal mucosa (*p* = 0.049 and *p* < 0.001, respectively). The PGI/PGII ratio best discriminated metaplasia and moderate atrophy (AUC 0.88 and 0.76, respectively). PGI and PGII alone showed poor discriminative ability, especially in mild atrophy (0.55 and 0.53, respectively) and chronic gastritis (0.55 and 0.53, respectively). The best cut-off to discriminate metaplasia was 3.25 U/mL (95.19% specificity, 83.33% sensitivity). Anti-*H. pylori* IgG and PG assessment (ABC method) was performed (group B, 2.0%; group A, 92.1%). The new cut-off more accurately identified patients with metaplasia requiring follow-up (group B, 5.4%). Assessment of anti-*H. pylori* IgG and PG is valuable in countries with a low prevalence of *H. pylori* infection.

## 1. Introduction

*Helicobacter pylori* is a rare pathogen that can successfully colonize the human stomach, and infection is an important factor related to the development of various pathological changes in the gastroduodenal tract [1,2]. *H. pylori* plays a major role in the development of chronic gastritis and peptic ulcers and is also associated with the development of gastric adenocarcinoma [1,3]. This organism is also responsible for gastric mucosal-associated lymphoid tissue lymphoma [4]. *H. pylori* is believed to infect half of the world’s population, and its prevalence has been reported to be notably higher in developing countries than in developed countries [5].

Previous studies conducted in the South Asian region have shown that the prevalence varies in different geographical locations. In India, the prevalence was reported to range from 58.8% to 85.0% [6]. A high prevalence was also reported in Bhutan and Bangladesh, where the rates were as high as 73.4% and 60.15%, respectively [7,8,9]. Sri Lanka is an island country in South Asia located in the Indian Ocean with a total population of approximately 21.6 million [10]. In previous studies, the prevalence of *H. pylori* infection in Sri Lanka ranged from 6.5% (12/184) in children by stool diagnosis to 70.1% (40/57) by PCR in 2002 [9,11]. A later study in 2015 reported that the *H. pylori* prevalence determined by PCR was 19.7% (15/76) [12]. The differences in the diagnostic methods used in each study and the low number of participants might have caused the wide range of prevalence.

Detection of *H. pylori* infection can be obtained via invasive and non-invasive measures. Detection of *H. pylori* through upper endoscopy, gastric biopsy, and culture provides evidence of infection [13,14]. It can also reveal the condition of the stomach and allow for the investigation of abnormal development of the gastric mucosa. However, these methods have numerous side effects and are expensive and time consuming. In addition, in the histological examination of a biopsy sample, only a small portion of the stomach is assessed.

The use of antibodies to detect *H. pylori* infection by serology has shown satisfactory results [15,16]. In a recent study of 2560 cases, among the non-invasive tests, *H. pylori* IgG detection showed the highest sensitivity (94%) compared to the urea breath test and stool antigen test (64% and 61%, respectively) using the histology results as a reference [17]. It is considered safe and sensitive for screening purposes. Countries with known high *H. pylori* prevalence, such as Japan, have determined a cut-off value of 10 U/mL [18]. However, the analysis of serology tests from many countries showed a variety of area under the curve (AUC) values [16,19]. Thus, validation and setting up a specific cut-off value for each country is necessary to optimize diagnostic performance [20].

Assessing pepsinogen (PG) levels is also a non-invasive approach for predicting gastric mucosa status [21,22]. PGI is produced by the oxyntic gland in the corpus, while PGII is also produced in the antrum, and the levels change during carcinogenesis [23]. PG levels increase in the presence of infection or inflammation but decrease in gland obliteration [24]. On the basis of this pathogenesis, researchers have proven that the changes in PGI level and PGI/PGII ratio are a good predictor for the development of gastric cancer in many countries such as Japan, Korea, and China [25,26,27,28]. In developing countries where endoscopy facilities are limited, such as in Sri Lanka, the PG test is advantageous for diagnostic and screening purposes. The combination of the PG test with *H. pylori* infection screening, which is known as the “ABC method”, has also been reported to be accurate and cost-effective [29,30]. Similar to other serology tests, this method also has different sensitivity and specificity ranges in each country [31,32,33] thus, validation is required before application to the general population.

It is important to investigate the burden of *H. pylori* infection. Eradication of *H. pylori* is mandatory to prevent gastroduodenal diseases. To our knowledge, this is the first study to validate the serology approach for the diagnosis of *H. pylori* and mucosal status in Sri Lanka. Hence, this study could help health policy makers in selecting the best strategy to reduce the burden of *H. pylori-*related diseases.

## 2. Material and Methods

### 2.1. Study Participants

The upper endoscopic survey was performed on 400 sequential subjects with dyspeptic symptoms at the University of Peradeniya and Teaching Hospital Peradeniya, Sri Lanka, from November 2017 to January 2018, including 204 males and 184 females with a mean age of 56.4 ± 14.7 and 57.9 ± 15.2 years, respectively. Dyspeptic patients aged >18 years without history of gastrectomy were included in this study. Any intake of proton pump inhibitors, antibiotics, or bismuth-containing compounds was prohibited 4 weeks prior to endoscopy.

Four biopsy specimens were collected during each endoscopy session, including two specimens from the greater curvature of the antrum, one specimen from the greater curvature of the corpus, and one specimen from the angulus. Biopsy from the antrum, corpus, and angulus was used for histopathological examination, while the remaining antrum specimens were used for *H. pylori* culture. For culture, antrum specimens were immediately placed in transport medium containing brucella broth (Becton Dickinson, Sparks, MD, USA) and glycerol (Nissui Pharmaceutical Co., Ltd., Tokyo, Japan) and stored at −80 °C until further use. Fasting serum was collected from each subject on the day of endoscopy and immediately stored at −20 °C until use.

All participants provided written informed consent, and study approval was obtained from the ethics committees of the University of Peradeniya, Sri Lanka, and Oita University Faculty of Medicine, Japan.

### 2.2. H. pylori Infection Status and Pepsinogen Level

*H. pylori* infection status was determined using three diagnostic tests: culture, histology, and serology. *H. pylori* culture was performed as described previously [34]. Briefly, the homogenized antrum specimens were inoculated onto *H. pylori-*selective media (Nissui Pharmaceutical Co., Ltd., Tokyo, Japan) and incubated for up to 10 days at 37 °C under microaerophilic conditions (10% O_2_, 5% CO_2_, and 85% N_2_). *H. pylori* was then sub-cultured onto Brucella agar (Becton Dickinson, Sparks, MD, USA) supplemented with 7% horse blood (Nippon Bio-test, Tokyo, Japan) without antibiotics. *H. pylori* was identified on the basis of colony morphology, Gram-negative staining results, bacterial morphology, and positive results for catalase (Sigma Aldrich, Darmstadt, Germany), urease (our laboratory homemade), and oxidase test (Milipore Sigma, Burlington, MA, USA). *H. pylori* was stored in Brucella broth (Becton Dickinson, Sparks, MD, USA) containing 10% glycerol and 10% horse serum and stored at −80 °C.

For histopathological examination, the biopsy materials were fixed in 10% formalin and embedded them in paraffin. Hematoxylin–eosin (Motu Chemical, Tokyo, Japan) and May–Grünwald–Giemsa (Merck, Darmstadt, Germany) staining were applied to thin slices of paraffin-embedded biopsy specimens. On the basis of the updated Sydney system, an experienced pathologist assessed the bacterial density in each specimen [34]. The neutrophils, monocytes, atrophy, and metaplasia were scored to determine the gastric mucosa status in the antrum, corpus, and angulus. The scoring system for the histology of gastric mucosa was also based on the updated Sydney system [35].

The definitions of each group are as follows: the normal group had a score of zero for all locations; the “chronic gastritis” group had a monocyte score of at least 1 without any atrophy or metaplasia, the “mild atrophic” group had an atrophy score of 1 without any metaplasia, the “moderate atrophic” group had an atrophy score of 2 without any metaplasia, and the “metaplasia” group had a metaplasia score of at least 1 in at least one location.

### 2.3. Anti-H. pylori IgG Test and PG Test from Serology

The serum antibody to *H. pylori* was examined using an E-Plate ELISA kit (Eiken Co., Ltd., Tokyo, Japan) following the manufacturer’s instructions. Participants with a serum anti-*H. pylori* level ≥10 U/mL were classified as *H. pylori-*positive. PGI and PGII levels were measured using a PG ELISA kit (Eiken, Tokyo, Japan) following the manufacturer’s instructions. The criteria by Miki et al., widely used in Japan, were applied to stratify the cancer risk; a PG I level ≤70 ng/mL and PG I/II ratio ≤3.0 were classified as PG-positive [29]. For further analysis, the group was determined on the basis of the serum anti-*H. pylori* and serum PG level using the ABC method: group A (*H. pylori*-negative/PG-negative), group B (*H. pylori*-positive/PG-negative), group C (*H. pylori*-positive/PG-positive), and group D (*H. pylori*-negative/PG-positive) [30].

### 2.4. Data Analysis

Statistical analyses were performed using R version 3.4.4. The ROC curve and Youden’s index were determined by the “pROC” package. The visualization and statistical comparison were performed using “ggplot”, “pubr”, and “ggsignif”. We analyzed the discrete variables using the chi-squared test, whereas continuous variables were analyzed using the Mann–Whitney *U* test, and statistical significance was defined as a *p*-value < 0.05.

## 3. Results

### 3.1. H. pylori Infection Status

A total of 400 subjects were recruited in this study. However, due to the incomplete biopsy and serum samples, 47 subjects were excluded. The final dataset of 353 subjects consisted of 164 females with an average age of 57.6 ± 15.0 years and 189 males with an average age of 56.5 ± 14.9 years. The predominant ethnicity of the subjects was Sinhalese (309/353; 87.5%), followed by Muslim (25/353; 7.1%) and Tamil (19/353; 5.4%).

First, a combination of culture and histology tests were used to determine the *H. pylori* infection status (Table 1). Six subjects had culture-positive results (6/353; 1.7%) and seven subjects had histology-positive results (7/370; 2.0%). With at least one test showing positivity by histology and culture, the percentage of *H. pylori* infection was 2.5% (9/353), consisting of six males and three females. Among the different ethnicities, Tamil had the highest infection rate (10.5%; 2/25) compared to Muslim (4.0%, 1/19) and Sinhalese (1.9%, 6/309).

### 3.2. Determination of H. pylori Infection Status by Anti-H. pylori IgG

The isolation of *H. pylori* bacteria in culture is a definitive approach showing current infection. However, the fastidious nature of *H. pylori* culture limits its feasibility in daily clinical practice. Histology evaluation showed 99.42% specificity, 57.14% sensitivity, and 98.58% accuracy in detecting *H. pylori* with the culture method as a reference.

**Figure 1:**
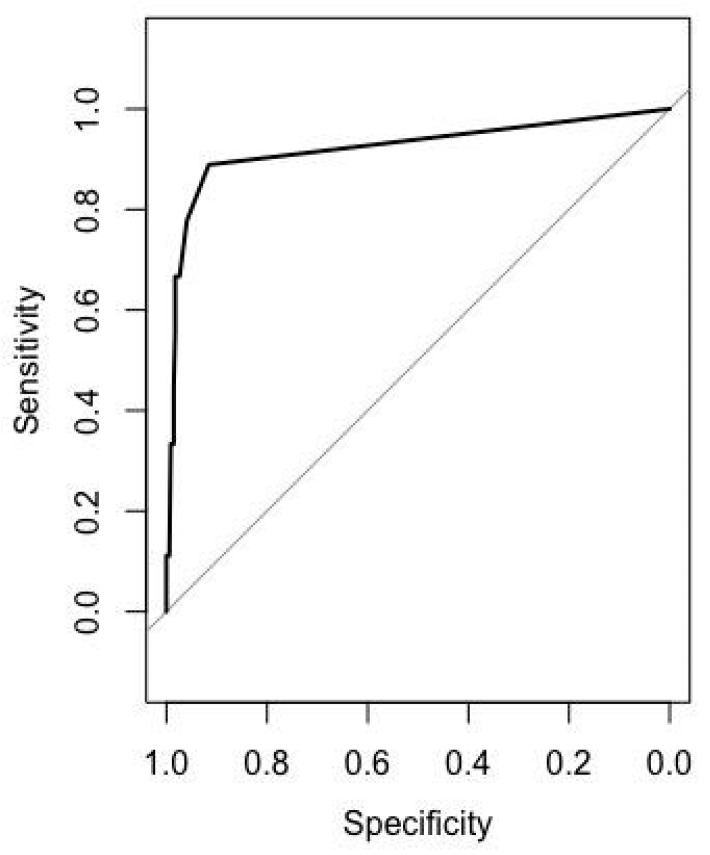
ROC curve of the *H. pylori* IgG to detect *H. pylori* infection.

The use of anti-*H. pylori* IgG in the serum to detect *H. pylori* infection has not been validated in Sri Lanka. Among all the samples, there was no significant correlation between *H. pylori* IgG status and age (*p* = 0.52, *r* = −0.03). The value of *H. pylori* IgG was significantly higher in *H. pylori*-positive patients (median, 6; IQR, 8.5; median, 2.9; IQR, 0.0; *p* value < 0.001). When the *H. pylori* IgG levels were plotted into the ROC (receiver operating characteristic) curve with the culture and histology results as the reference, the AUC was 0.922 (Figure 1). In the ABC method, the cut-off value for *H. pylori* infection was 10.0 U/mL. However, when the cut-off was applied to the Sri Lankan population, the sensitivity was poor (33.33%), even though the specificity was 98.27%. Therefore, in the process of setting a new cut-off for *H. pylori* infection, we achieved the best sensitivity and specificity (91.6% and 88.9%, respectively) at a value of 2.95 U/mL. The positive likelihood ratio, negative likelihood ratio, positive predictive value, and negative predictive value of the new cut-off were 18.07, 0.17, 23.7, and 99.7%, respectively. The seroprevalence of *H. pylori* IgG (2.95 U/mL) was 6.2% (22/353).

### 3.3. Endoscopy Diagnosis and Histology Examination

Upper endoscopy revealed gastric ulcers and duodenal ulcers in 7.4% (26/353) and 1.1% (4/353) of the subjects, respectively. Gastritis (65.7%; 232/353) and normal-appearing mucosa on endoscopy (25.8%; 91/353) were predominant in this study. However, among the gastric ulcer patients, only 19.2% (5/26) were infected with *H. pylori,* and in the duodenal ulcer patients, only 25% (1/4) were infected.

**Figure 2:**
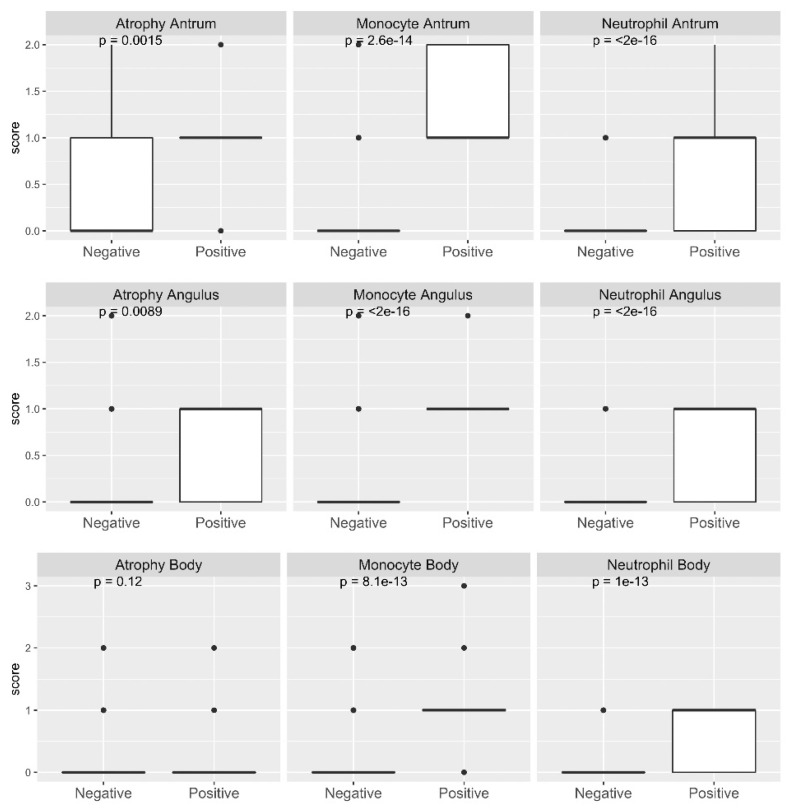
Distribution of histology assessment results among *H. pylori-*positive and -negative samples. A statistically significant difference in neutrophil, monocyte, and atrophy scores between the *H. pylori-*positive and -negative subjects was observed in the antrum, angulus, and body (*p* < 0.001) according to at least one positive result from several diagnostic methods. Only one subject was reported to have a metaplasia.

From the histological examination, the score of the severity of inflammation was based on the presence of neutrophils, monocytes, atrophy, and intestinal metaplasia in the antrum, corpus, and angle (Figure 2). The neutrophil and monocyte scores in all locations ranged from 0 to 3, but the scores for atrophy and metaplasia ranged from 0 to 2; there were no patients with severe atrophy and/or metaplasia. The median value and difference among *H. pylori* infection status based on the serology test (*H. pylori-*positive patients = 22) are shown in Figure 2. *H. pylori* infection significantly increased the scores of neutrophils, monocytes, atrophy, and metaplasia. The scores obtained by histology were also used to classify the subjects into five categories according to their severity. Among the 94.8% (331/353) of the *H. pylori-*negative subjects, the bacteria could not be detected by histology and culture in four subjects, but the severity of gastric mucosal inflammation showed moderate atrophy or metaplasia (Table 2). This phenomenon could have occurred because of past infection.

### 3.4. Analysis of Serum PG and Gastric Cancer Risk Determination by the ABC Method

**Figure 3:**
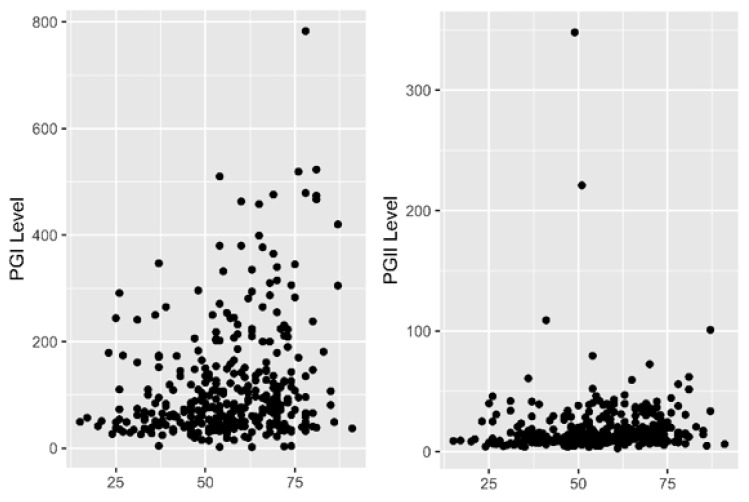
Correlation of pepsinogen (PG) with age. *X*-axis represents the age and *Y*-axis represents levels. PGI level and PGI/PGII ratio were weakly correlated with age. PGII was not significantly correlated with age.

PG levels from the serum could reflect the gastric mucosa status, indicating whether patients have chronic inflammation atrophy or metaplasia. It is also known as a non-invasive approach for identifying the risk of gastric cancer development. Hence, PGI and PGII levels were measured in 353 subjects. The average PGI levels are shown in Table 3. The PGI and PGII levels were significantly higher in male subjects than in female subjects (*p* = 0.002 and *p* = 0.009, respectively). For *H. pylori* infection status, the PGI/PGII ratio was significantly lower in *H. pylori*-positive patients than in *H. pylori*-negative patients (*p* < 0.001) (Table 3). The PGI level and PGI/PGII ratio were positively correlated with age, although the relationship was weak (*p* < 0.0001 and 0.003; *r* = 0.29 and 0.17, respectively) (Figure 3).

**Figure 4:**
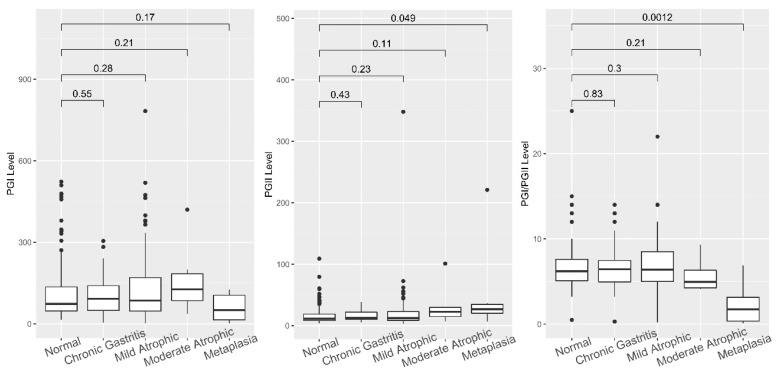
PGI, PGII, and PGI/PGII ratio among the different histology status groups. The PGI levels among groups were not significantly different. However, a significant difference in PGII and PGI/PGII ratio was observed between the normal and metaplasia groups.

The PG level was analyzed among the different gastric mucosa statuses according to histological evaluation. The subjects were then classified into five groups according to their scores, as shown in Figure 4. PGII increased with the deterioration of mucosa (*p* = 0.043, r = 0.11) and significantly increased in the presence of metaplasia compared to normal tissue (*p* = 0.049). Unlike PGII, the PGI and PGI/PGII ratio did not have a significant correlation with the severity of mucosa deterioration (*p* = 0.3, r = 0.05, and *p* = 0.84, r = −0.01, respectively). In contrast, the PGI/II ratio in the metaplasia group was significantly lower than that of the normal group (*p* = 0.0012).

### 3.5. Diagnostic Value of Pepsinogen to Discriminate Gastric Mucosa Status

The ABC method utilizes a cut-off value for PGI of 10.0 and a PGI/PGII ratio of greater than or equal to 3. This cut-off supposedly discriminates early gastric cancer. To clarify the histological stage of early gastric cancer that can be distinguished by PGI and PGI/PGII ratio according to the criteria by Miki [30], we evaluated the sensitivity, specificity, positive likelihood ratio, and negative likelihood ratio with the gastric mucosa histology status as the reference. A new cut-off value was then identified to improve the diagnostic performance of PG to predict histological status.

According to the criteria stated by Miki et al., PGI positivity was defined as surpassing 70 U/mL with a PGI/PGII ratio > 3.0. The sensitivity and specificity analysis of PGI and PGI/PGII ratio to predict the presence of chronic gastritis, mild atrophy, moderate atrophy, and metaplasia are shown in Table 4. Using this cut-off, the specificity of the PGI/PGII ratio to determine chronic gastritis and mild and moderate atrophy was excellent (99.5%, 99.1%, and 97.07%, respectively), but the sensitivity was extremely poor (8.38%, 9.02%, and 33.3%, respectively). Therefore, the accuracy was low. However, the performance of the PGI/PGII ratio was improved when it was applied to discriminate the presence of metaplasia with a sensitivity of 66.67%, specificity of 97.12%, and accuracy of 96.6%.

**Figure 5:**
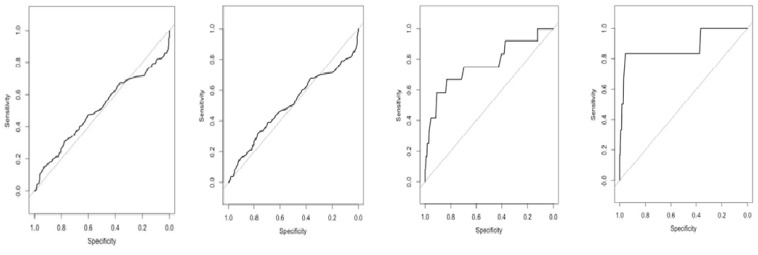
ROC curve of PGI/PGII ratio. From left to right, AUC curve analysis of PGI/PGII ratio for chronic gastritis, mild atrophy, moderate atrophy, and metaplasia. The highest AUC was 0.8804 to discriminate the presence of metaplasia.

To further define the diagnostic values of PGI, PGII, and the PGI/PGII ratio that are more suitable for application in the Sri Lankan population, we performed ROC curve analysis and determined the new cut-off value by Youden’s index. As depicted in Table 5 and Figure 5, the ROC analysis of PGI, PGII, and the PGI/PGII ratio showed poor discrimination for chronic gastritis and mild atrophy (AUC around 0.5). However, the AUC of PGII and the PGI/PGII ratio to discriminate moderate atrophy increased to 0.70 and 0.76, respectively. The highest AUC was obtained when the PGI, PGII, and PGI/PGII ratio were used to determine metaplasia (0.68, 0.72, and 0.88, respectively). The new cut-off value could improve the sensitivity of PGI, PGII, and the PGI/PGII ratio to determine chronic gastritis, mild and moderate atrophy, and metaplasia. The PGI/PGII ratio is the best candidate for determining moderate atrophy and metaplasia (specificity 83.28% and 95.39%, sensitivity 66.67% and 83.33%, respectively).

### 3.6. Diagnostic Value of PG to Discriminate Gastric Mucosa Status

We evaluated the use of PG to predict and differentiate gastric mucosa status. Using the criteria reported by Miki that have been widely used in Japan by using the data of PGI, PGI/PGII ratio, and *H. pylori* infection status by anti-*H. pylori* IgG, we classified the subjects into groups A to D, as shown in Table 5. The majority of the subjects (94.9%; 335/353) belonged to group A, which had the lowest risk of gastric cancer. Groups B, C, and D comprised 2.0% (7/353), 0.2% (1/353), and 2.8% (10/353) of patients, respectively. From this result, in group A, the normal histology was predominant (58.8%, 197/335), although this group also had a high proportion of mild atrophy (33.1%, 111/335). Among all groups, group D had the highest risk of requiring intervention and surveillance.

As shown in Table 6, the modification of the criteria could capture more moderate atrophy and metaplasia in groups B and C. The moderate atrophy and metaplasia required follow-up to diagnose the development of gastric cancer early; thus, they should not be misclassified into group A.

## 4. Discussion

This study included the largest number of subjects among the studies performed in Sri Lanka. According to the histology and culture results, the prevalence of *H. pylori* infection was only 1.69% (7/353). A previous study reported the prevalence in Sri Lanka using the stool antigen test and PCR [9,11,12]. A recent systematic review that compared the accuracy of diagnostic tests ranked the quality according to the superiority index range from 2.17 to 9.94 [13]. For the PCR and stool antigen, the highest superiority index was only 4.99 for the PCR and 3.87 for the stool antigen. The superiority index was lower than that of histology, culture, and serology (7.51, 7.35, and 7.81, respectively). Another study also mentioned that culture was considered to have 100% specificity with a specificity of approximately 85% despite the fastidious nature of *H. pylori* growth [36]. In the absence of a gold standard, a combination of different methods can be used to detect *H. pylori* to compensate for the sensitivity and specificity. This study attempted to confirm these results using several diagnostic methods. The first step is to validate the serology test using IgG on the basis of histology and culture.

Our results showed that *H. pylori* infection was poorly detected using anti-H. *pylori* IgG with a cut-off of 10 U/mL, as determined by the manufacturer’s instructions [30,37,38]. These criteria have been used in Japan for screening [29]. However, in Sri Lanka, these criteria showed a poor sensitivity (33.3%); thus, a new cut-off of 2.95 U/mL was determined, which improved the sensitivity, specificity, and accuracy. Using the new cut-off, the prevalence of *H. pylori* according to serology was 6.2% (22/353). Among serology-positive subjects, *H. pylori* in four subjects could not be detected by histology and culture, but histological examination showed moderate atrophy and metaplasia. This may indicate past infections [39]. A previous study reported that in patients with a high titer of anti-*H. pylori* IgG, 17.5% of them were previously infected, and the infection had been eradicated [40]. In addition, the current cohort study showed that IgG titers between 3 and 10 U/mL also indicated a higher risk for gastric cancer [40].

Sri Lanka is isolated by the ocean from the mainland of other South Asian countries. As recorded in this study, the subjects predominantly belonged to the native Sinhalese ethnic group. Despite the high prevalence of *H. pylori* infection in other South Asian countries such as India (81%), Nepal (28.9–38.4%), and Bangladesh (42.1%), Sri Lanka had an extremely low prevalence of *H. pylori* infection [41,42,43]. This result provides evidence that *H. pylori* prevalence could vary according to geographical location, indicating the strong roles played by the host and environment in the susceptibility to infection. Only a few countries have reported extremely low prevalence of *H. pylori*. Malaysia and some regions in Indonesia have also been reported as low-prevalence countries with low gastric cancer risk [34,44,45]. Hence, the low prevalence of *H. pylori* infection could be attributed to the low age standardized rate of gastric cancer in Sri Lanka.

Histological examination revealed a significantly higher inflammatory and atrophy status in *H. pylori*-positive cases than in *H. pylori*-negative cases. Gastric atrophy is the heavy deterioration of gland function in the antrum or corpus, which soon turns into metaplasia [46,47]. Even though the prevalence is considered low, the detection of *H. pylori* infection must not be neglected in chronic gastritis cases, and it requires immediate eradication [48]. Despite the low prevalence of *H. pylori* infection, histological evaluation in Sri Lanka showed a high proportion of mild atrophy (34.3%, 121/323) and 1.7% (6/353) moderate atrophy. Among the gastric and duodenal ulcer groups, the proportion of patients infected with *H. pylori* was also low. These phenomena could be related to other causes, such as consumption of NSAIDs, use of proton pump inhibitors, autoimmune gastritis, and alteration of microbiome composition in the stomach [22,49,50].

For the examination of the gastric mucosa status, assessment of biomarkers such as PG could be an alternative to the histology method [51]. Although the PG value showed a positive correlation with age, the coefficient was low, as mentioned in several previous studies [52,53]. The higher level of PG in *H. pylori-*positive patients due to inflammation reaction is concordant with previous studies [27,54]. From the analysis of PG considering the inflammation severity of the gastric mucosa, PGI levels significantly decreased in the presence of metaplasia, while PGII levels increased. This phenomenon results in a significantly low PG/PGII ratio [55,56]. From the ROC analysis, the PGI level appeared to have the highest discriminatory ability in determining metaplasia among all gastritis statuses (Table 5). It could be a valuable diagnostic method in the absence of endoscopy facilities, as well as an indicator of high risk and therefore contraindication for endoscopy.

The combination of *H. pylori* status and PGs (known as the ABC method) has been applied in Japan and other countries as a screening method for gastric cancer [30]. The ABC method has criteria that were supported by a cohort study [40,57]. However, the use of a value of 10 U/mL as the cut-off seems to omit patients with moderate atrophy and severe metaplasia. Severe metaplasia should be followed by endoscopy [30]. Utilizing the best cut-off for the PGI/PGII ratio improved the specificity, sensitivity, and accuracy. The PGI/PGII ratio is recommended for use because it has the highest diagnostic performance compared to PGI and PGII alone. According to the carcinogenesis cascade, atrophy is considered a reversible state, while metaplasia has little chance of returning to normal [46,58]. Early detection by screening is an effective method for preventing cancer [48]. Hence, for gastric cancer screening purposes, the earlier the precursor of the disease is detected, the more beneficial it is for patients [59]. As shown in Table 5 and Figure 3, the highest AUC was achieved in metaplasia followed by moderate atrophy. PGI/PGII can also detect moderate atrophy with 83.3% specificity and 66.7% sensitivity. This result suggests the potential implementation of the PG test for detection of *H. pylori* infection and early inflammation of the gastric mucosa in Sri Lanka [60].

## 5. Conclusions

This study revealed the extremely low prevalence of *H. pylori* infection in Sri Lanka according to three diagnostic methods. However, infection still causes significant damage to the stomach histopathology; thus, effective diagnosis and eradication of *H. pylori* remains a necessity. In this setting, serology IgG proved its diagnostic value in detecting *H. pylori.* PG could also be an effective alternative marker to determine gastric mucosa status, especially metaplasia, along with *H. pylori* IgG.

## Figures and Tables

**Table 1:** *H. pylori* infection rate by histology and culture.

Subject Characteristics	Total Subjects	Positive Result (%)		
		Culture	Histology	Culture and Histology
***n* test positive**	353	6 (1.6)	7 (1.9)	9 (2.5)
**Sex**				
Male	189	4 (2.1)	5 (2.6)	6 (3.2)
Female	164	2 (1.2)	2 (1.2)	3 (1.8)
*p*-value		0.68	0.46	0.51
**Ethnicity**				
Sinhalese	309	4 (1.3)	5 (1.6)	6 (1.9)
Muslim	25	1 (4.0)	1 (4.0)	1 (4.0)
Tamil	19	1 (5.2)	1 (5.2)	2 (10.5)
*p*-value		0.16	0.21	0.06

**Table 2:** Gastric mucosal status of patients.

	Histology Culture-Negative		Histology Culture-Positive	
	*H. pylori-*IgG Positive	*H. pylori-*IgG Negative	*H. pylori-*IgG Positive	*H. pylori-*IgG Negative
Normal	2	196	0	0
Chronic gastritis	1	20	1	0
Mild atrophy	7	108	5	1
Moderate atrophy	2	3	0	1
Metaplasia	2	3	1	0
Total	14	330	7	2

**Table 3:** Pepsinogen level by *H. pylori* infection status and gender.

	PGI	PGII	PGI/PGII
All sample summary			
Median	77.9	12.2	6.3
Min	2	2.6	0.1
Max	783	348	25
IQR	98.5	12.4	2.8
*H. pylori* infection status (median; IQR)			
Positive (*n* = 23) *	69.9; 74.1	16.9; 17.5	4.1; 1.8
Negative (*n* = 339)	78.5; 99.1	12; 12.05	6.3; 2.9
*p*-value	0.31	0.19	<0.001
Gender (median; IQR)			
Male (*n* = 189)	92.8; 109.8	13.7; 16.7	6.3; 2.7
Female (*n* = 164)	68.2; 111.3	11.2; 18.3	6.1; 7.7
*p*-value	0.0018	0.0089	0.28

* *H. pylori-*positive according to at least one positive result (histology, culture, or serology).

**Table 4:** Diagnostic value of the Miki criteria to predict gastric mucosa status.

Parameters	Chronic Gastritis		Mild Atrophy		Moderate Atrophy		Metaplasia	
	PGI	PGI/PGII Ratio	PGI	PGI/PGII Ratio	PGI	PGI/PGII Ratio	PGI	PGI/PGII Ratio
True positive	64	13	56	12	4	4	3	4
True negative	104	197	118	218	187	331	192	337
False positive	94	1	102	2	154	10	155	10
False negative	91	142	77	121	8	8	3	2
Sensitivity (%)	41.29	8.38	42.11	9.02	33.33	33.33	50	66.67
Specificity (%)	52.53	99.5	53.64	99.1	54.84	97.07	55.33	97.12
Positive likelihood	0.89	16.61	0.91	9.93	0.74	11.37	1.12	23.13
Negative likelihood	1.18	0.93	1.08	0.92	1.21	0.69	0.9	0.3432
PPV	40.5	92.85	49.39	85.73	2.52	28.51	1.08	28.45
NPV	53.34	58.12	46.3	64.28	95.9	97.65	98.47	99.41
Accuracy	47.59	59.49	49.29	65.16	54.11	94.9	55.24	96.6

**Table 5:** Analysis of ROC and cut-off value.

	AUC	New Cut-Off	Specificity (%)	Sensitivity (%)	Control	Case	*p*-Value
Chronic gastritis							
PGI	0.5335	92.45	62.12	48.38	198	155	0.28
PGII	0.5552	17.45	71.22	40.64			0.07
PGI/PGII ratio	0.5081	9.75	93.43	14.83			0.79
Mild atrophy							
PGI	0.529	92.45	60.91	48.12	220	133	0.36
PGII	0.5517	17.45	71.82	42.11			0.1
PGI/PGII ratio	0.5055	7.65	76.36	33.08			0.86
Moderate atrophy							
PGI	0.4785	111	66.28	50	341	12	0.8
PGII	0.6987	14.55	60.12	83.33			0.019
PGI/PGII ratio	0.7652	4.75	83.28	66.67			0.0017
Metaplasia							
PGI	0.6804	15.15	98.84	50	347	6	0.13
PGII	0.7207	18.15	69.16	83.33			0.064
PGI/PGII ratio	0.8804	3.25	95.39	83.33			0.0014

**Table 6:** Gastric mucosa status of each group using the ABC method with the Miki criteria versus the Sri Lanka-modified criteria.

		**ABC Method According to Criteria by Miki, *n***			
	**Total**	**A**	**B**	**C**	**D**
Number in each group	353	335	7	1	10
Histology of gastric tissue					
Metaplasia	6	2	1	0	3
Moderate atrophic	6	4	2	0	0
Mild atrophic	121	111	4	1	5
Chronic gastritis	22	21	0	0	1
Normal	198	197	0	0	1
		**Sri Lanka-modified criteria, *n***			
	**Total**	**A**	**B**	**C**	**D**
Number in each group	353	323	19	3	8
Histology of gastric tissue					
Metaplasia	6	1	2	1	2
Moderate atrophic	6	4	2	0	0
Mild atrophic	121	104	11	2	4
Chronic gastritis	22	19	2	0	1
Normal	198	195	2	0	1

## Data Availability

The data presented in this study are available on request from the corresponding author.
